# Inhibition of autophagy potentiates pemetrexed and simvastatin-induced apoptotic cell death in malignant mesothelioma and non-small cell lung cancer cells

**DOI:** 10.18632/oncotarget.5022

**Published:** 2015-08-20

**Authors:** Ki-Eun Hwang, Young-Suk Kim, Jae-Wan Jung, Su-Jin Kwon, Do-Sim Park, Byong-Ki Cha, Seon-Hee Oh, Kwon-Ha Yoon, Eun-Taik Jeong, Hak-Ryul Kim

**Affiliations:** ^1^ Department of Internal Medicine, Institute of Wonkwang Medical Science, Wonkwang University, School of Medicine, Iksan, Jeonbuk, Korea; ^2^ Department of Laboratory Medicine, Wonkwang University, School of Medicine, Iksan, Jeonbuk, Korea; ^3^ Department of Thoracic and Cardiovascular Surgery, Chonbuk National University Medical School, Jeonju, Jeonbuk, Korea; ^4^ Department of Natural Medical Sciences, College of Health Science, Chosun University, Seosuk-dong, Gwangju, Korea; ^5^ Department of Radiology, Wonkwang University School of Medicine, Iksan, Jeonbuk, Korea

**Keywords:** autophagy, apoptosis, pemetrexed, simvastatin

## Abstract

Pemetrexed, a multitarget antifolate used to treat malignant mesothelioma and non-small cell lung cancer (NSCLC), has been shown to stimulate autophagy. In this study, we determined whether autophagy could be induced by pemetrexed and simvastatin cotreatment in malignant mesothelioma and NSCLC cells. Furthermore, we determined whether inhibition of autophagy drives apoptosis in malignant mesothelioma and NSCLC cells. Malignant mesothelioma MSTO-211H and A549 NSCLC cells were treated with pemetrexed and simvastatin alone and in combination to evaluate their effect on autophagy and apoptosis. Cotreatment with pemetrexed and simvastatin induced greater caspase-dependent apoptosis and autophagy than either drug alone in malignant mesothelioma and NSCLC cells. 3-Methyladenine (3-MA), ATG5 siRNA, bafilomycin A, and E64D/pepstatin A enhanced the apoptotic potential of pemetrexed and simvastatin, whereas rapamycin and LY294002 attenuated their induction of caspase-dependent apoptosis. Our data indicate that pemetrexed and simvastatin cotreatment augmented apoptosis and autophagy in malignant mesothelioma and NSCLC cells. Inhibition of pemetrexed and simvastatin-induced autophagy was shown to enhance apoptosis, suggesting that this could be a novel therapeutic strategy against malignant mesothelioma and NSCLC.

## INTRODUCTION

Non-small cell lung cancer (NSCLC) accounts for approximately 80% of all lung cancers, with long-term survival restricted to a small patient subset. The majority of NSCLC patients show locally advanced, inoperable, or metastatic diseases [1], and cytotoxic chemotherapies have had a modest but significant impact on survival and quality of life of NSCLC patients [2]. Malignant mesothelioma is a relatively rare malignancy with a generally poor outcome. Currently, the median survival is 9–12 months after diagnosis. At present, there are few effective chemotherapeutic options for malignant mesothelioma [3]. New strategies based on a better understanding of tumor biology may help maximize the efficacy of current treatments.

Autophagy is a dynamic process in which intracellular membrane structures sequester proteins and organelles for degradation in a lytic compartment [4, 5]. During autophagy, parts of the cytoplasm are sequestered into double-membrane vesicles called autophagosomes. Autophagosomes ultimately fuse with lysosomes to generate single-membrane autophagolysosomes that mediate the degradation of their contents [6]. A number of stimuli can induce autophagy, apoptosis, or both; with concomitant induction, autophagy can either protect against, or promote apoptosis in a cell stimulus-dependent manner [7, 8]. The molecular mechanisms that determine autophagy, apoptosis, and their interaction are not fully established, but may involve the induction of autophagy genes such as Atg5 in a cell type-, stimulus-, and cellular environment-specific manner.

Pemetrexed, a multitargeted antifolate antagonist, is a chemotherapeutic agent used in the treatment of malignant mesothelioma and NSCLC (mostly used in nonsquamous cell carcinomas) [9–11]. It primarily inhibits thymidylate synthase (TS), dihydrofolate reductase (DHFR), and glycinamide ribonucleotide formyltransferase (GARFT), all of which are enzymes involved in folate-dependent metabolic processes [12, 13]. Previous studies have reported that pemetrexed-induced apoptosis is associated with the upregulation of p53 and inactivation of Bcl-2 [14, 15]. Additionally, inhibition of the intrinsic apoptosis pathway has been shown to suppress the cytotoxicity of pemetrexed [16]. Dent et al. [17] demonstrated that pemetrexed induces tumor cell death and autophagy in a dose-dependent fashion.

Statins are a class of drugs that inhibit the rate-limiting step of the mevalonate pathway, which is catalyzed by 3-hydroxy-3-methylglutaryl coenzyme A (HMG-CoA) reductase [18]. Besides their lipid-lowering effect, statins have been studied for their antineoplastic properties in many solid tumor cells, including NSCLC [19, 20]. We have demonstrated previously that simvastatin induced apoptosis in lung cancer via AKT-dependent downregulation of survivin [21]. A recent study has also revealed that statins induce autophagy that is dependent on AMP-activated protein kinase (AMPK) activation [22]; however, the precise mechanism for this effect has not been clearly elucidated.

Although pemetrexed has generally been a well-tolerated drug, its toxicity profile is not trivial. The most frequently observed adverse effects include myelosuppression, fatigue, hepatotoxicity, nephrotoxicity, pneumonitis, and mucositis [23, 24]. Until recently, the mechanism by which a combination of pemetrexed and statins induced apoptosis and inhibited the growth of cancer cells had not been elucidated. We recently showed that cotreatment with pemetrexed and simvastatin potentiates apoptotic activity over that seen with either drug alone in malignant mesothelioma and lung cancer cells. These effects were mediated through mitochondrial dysfunction, by triggering reactive oxygen species (ROS) production and Bim induction [25].

In this study, we hypothesized that autophagy plays an important role in the outcome of apoptosis during pemetrexed and simvastatin treatment. We analyzed the coordinating mechanisms mediating the interplay between these processes in malignant mesothelioma and NSCLC cells.

## RESULTS

### Effect of pemetrexed and simvastatin alone and in combination, on the proliferation and apoptosis of malignant mesothelioma and NSCLC cells

**Figure 1:**
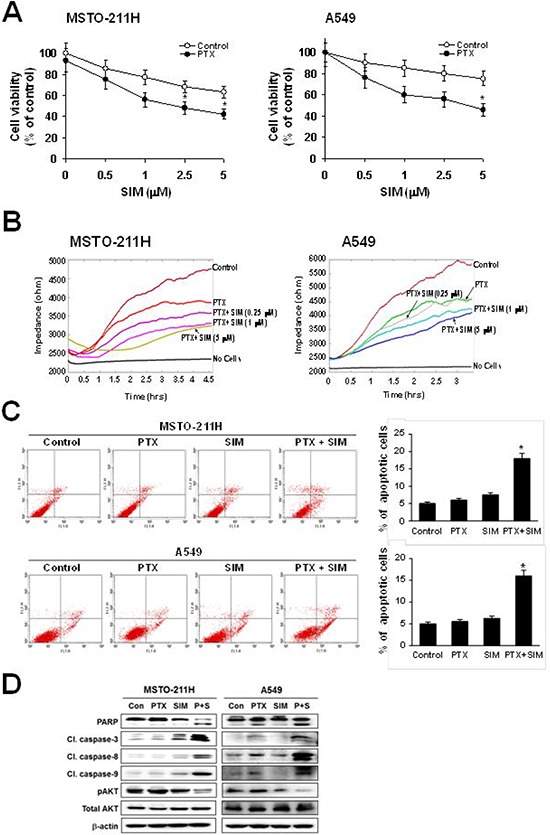
Inhibitory effects of pemetrexed and simvastatin cotreatment on the proliferation and apoptosis **A.** Cells were treated with different concentrations of simvastatin (SIM) in the absence or presence of 1 μM pemetrexed (PTX) for 48 h, and viability was then measured by the MTT assay. The viability of control cells was set at 100%, and the cell survival of treated cells relative to the control cells is presented. The data represent the mean ± S.D. of three independent experiments. *p < 0.05 compared to the control. **B.** Cells were treated with different concentrations of simvastatin in the presence of 1 μM pemetrexed. Proliferation analysis was performed using the electrical cell-substrate impedance sensing system (ECIS). Resistance was measured at 6400 Hz every 10 minutes for a period of 48 hours. During the experiments, cultures were maintained at 37°C and 5% CO_2_ in air. **C.** Cells were incubated with 1 μM pemetrexed and/or 5 μM simvastatin for 24 h, and apoptosis was evaluated by green fluorescent protein-annexin V + propidium iodide. The percentage of annexin V and propidium iodide positive cells in control cells was set at 100%, and the percentage of apoptosis relative to that of the control is presented. The data represent the mean ± SD of three independent experiments. *p < 0.05 compared to control. **D.** Cells were treated with pemetrexed and simvastatin, alone and in combination for 24 h. Then the cells were lysed, and the cell lysate was subjected to 12% SDS-PAGE to measure the expression of the indicated proteins. Data are representative of two independent experiments.

As shown in Figure 1A, pemetrexed and simvastatin cotreatment produced a addictive effect on the growth of MSTO-211H and A549 cells. In the presence of pemetrexed, simvastatin inhibited viability in a concentration-dependent manner. Using ECIS, we quantitatively evaluated cell proliferation. In this assay, an increase in resistance reflects cell proliferation. The ECIS data (Figure 1B) showed that the pemetrexed and simvastatin cotreatment suppressed cell proliferation in a dose-dependent fashion.

To examine whether the observed growth inhibition was due to enhanced apoptosis, the proportion of apoptotic cells was determined using annexin V-PI staining. Annexin V staining showed that combination treatment significantly enhances apoptosis compared to either drug alone in MSTO-211H and A549 cells (Figure 1C). To further elucidate the mechanism behind pemetrexed- and simvastatin-induced apoptosis, cell lysates were evaluated by immunoblotting (Figure 1D). Our results showed that the pemetrexed and simvastatin cotreatment enhanced the cleavage of PARP, caspase-3, -8, and -9. Additionally, AKT phosphorylation was significantly attenuated in MSTO-211H and A549 cells treated with pemetrexed and simvastatin. These results indicate that these drugs enhance caspase-dependent apoptosis in MSTO-211H and A549 cells.

### Pemetrexed and simvastatin cotreatment enhances autophagy in malignant mesothelioma and NSCLC cells

**Figure 2:**
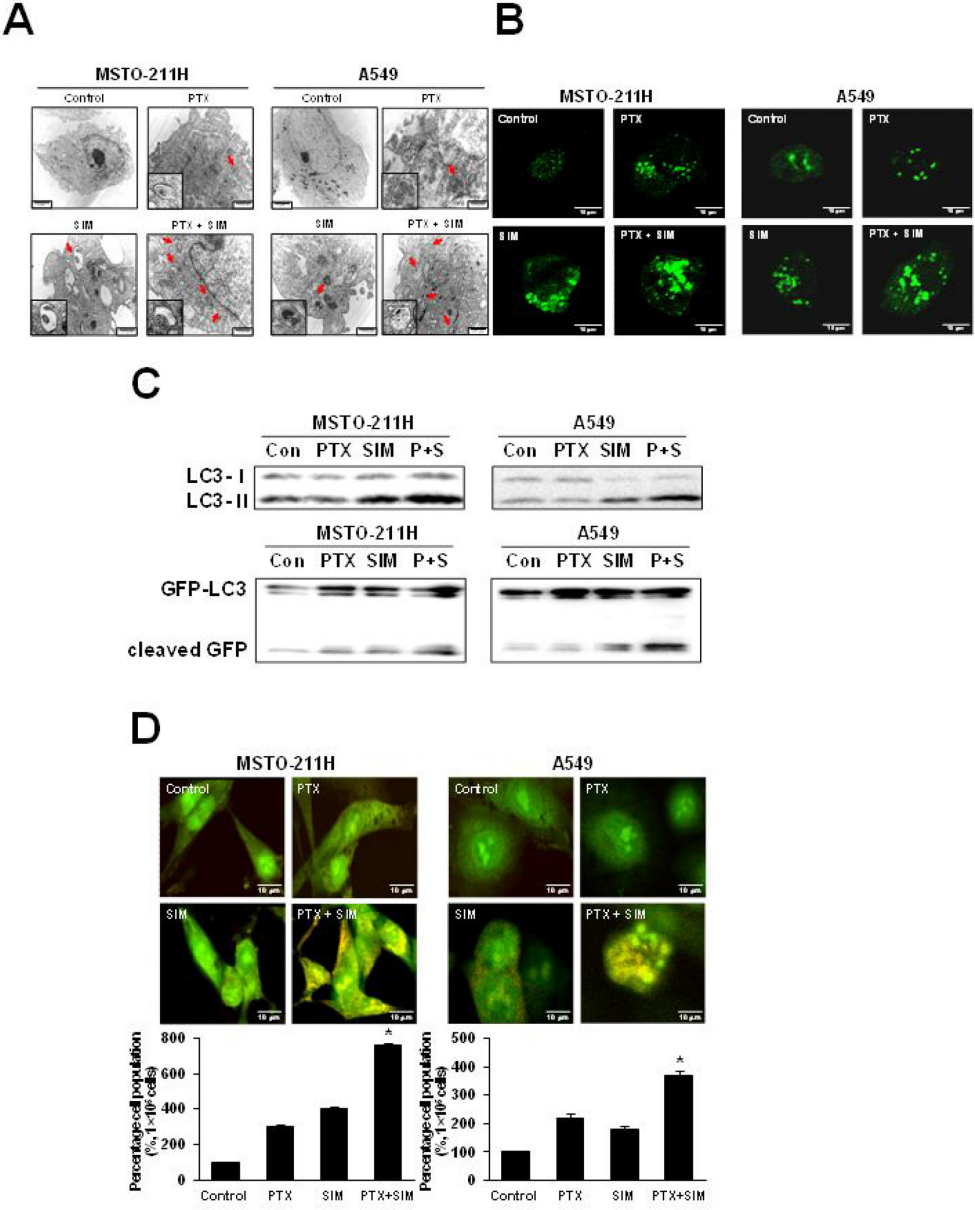
Combination of pemetrexed and simvastatin enhances autophagy in MSTO-211H and A549 cells **A.** Representative transmission electron microscopy photographs of cells treated with 1 μM pemetrexed and/or 5 μM simvastatin for 24 h (×10,000). Structures identified as autophagosomes are indicated with arrows. Autophagosomes are highlighted in magnified images of each cytosolic vesicle. **B.** Cells were transfected with the GFP-LC3 plasmid for 6 h and then incubated with 1 μM pemetrexed and/or 5 μM simvastatin for 24 h before analysis by confocal microscopy. Representative images of GFP-LC3 stained pemetrexed and/or simvastatin treated cells are shown (×400). A punctuate pattern of GFP-LC3-II indicates autophagosome formation, as shown by confocal microscopy. **C.** We performed western blot analysis using antibodies against endogenous LC3 and GFP. Immunoblots are representative of at least two independent experiments. **D.** Acridine orange staining showed lysosomal (orange) staining in the cells of all treatments. The increased acidic lysosomes in the combination treatment suggest potential lysosomal activation. The percentage of lysosomal (orange) stained cells was quantified. The data represent the mean ± SD of three independent experiments. *p < 0.05 compared to control.

Because autophagy and apoptosis may occur concurrently or sequentially in response to the same stimulus, we analyzed the cellular ultrastructure by TEM to confirm that autophagy was induced by pemetrexed and simvastatin. The combination treatment led to the formation of numerous lipid droplets, shown as hollow cytoplasmic vesicles and lamellar bodies, a hallmark of phospholipidosis. Furthermore, multiple autophagosomes containing cytoplasmic components were observed in MSTO-211H and A549 cells treated with both pemetrexed and simvastatin (Figure 2A).

Autophagic induction by combination of pemetrexed cotreatment and simvastatin was further confirmed by transient transfection of green fluorescence protein (GFP)-LC3-II plasmid DNA. In non-treated control cells, a diffuse pattern of GFP fluorescence was observed in the cytoplasm; however, MSTO-211H and A549 cells treated with both pemetrexed and simvastatin displayed markedly more LC3-positive GFP punctae in compared to cells treated with pemetrexed or simvastatin alone (Figure 2B).

In both cell lines tested, we found that a combination of pemetrexed and simvastatin induced a greater conversion of LC3-I into LC3-II than individual drugs. Autophagic induction was also determined by detecting free GFP fragments formed by GFP-LC3 degradation in autophagolysomes. GFP generation showed that autophagic induction was significantly increased by pemetrexed and simvastatin combination compared to individual treatment with either drug (Figure 2C).

Finally, autophagic detection was also evaluated using lysosomal staining with acridine orange dye and observed under an inverted microscope to measure fluorescence. Both pemetrexed and simvastatin alone slightly increased acidic lysomal compartments, while an increase in acridine orange intensity was highly apparent in the combined treatment, as shown by FACS analysis (Figure 2D).

### Pemetrexed and simvastatin cotreatment induces AMPK- and AKT-mediated mTOR-dependent autophagy

**Figure 3:**
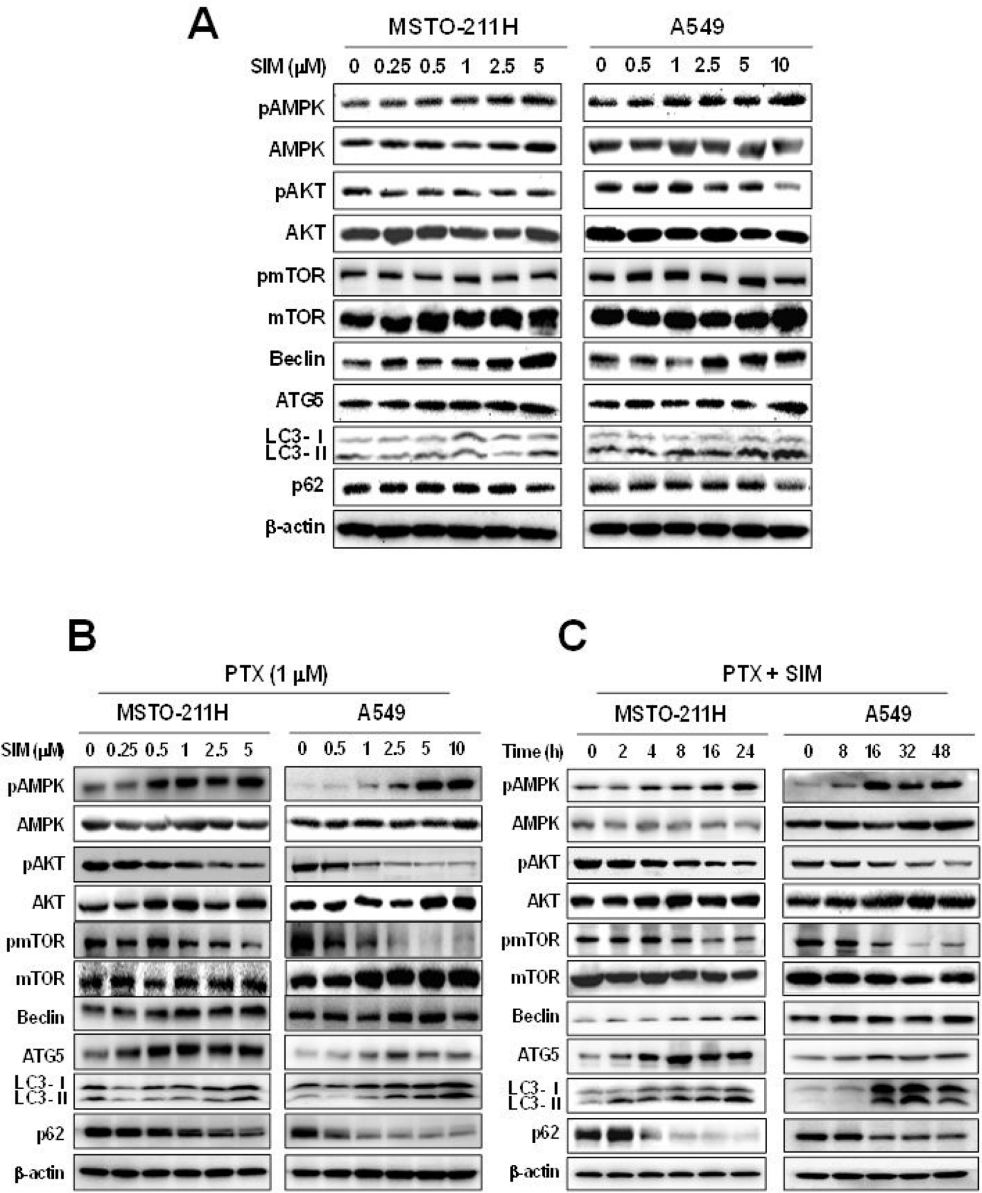
Combination of pemetrexed and simvastatin induces autophagy through the mTOR-signaling pathway Cells were treated with different concentrations of simvastatin alone **A.** or 1 μM pemetrexed **B.** or were incubated with 1 μM pemetrexed and 5 μM simvastatin for up to 48 h **C.** and Western blot analysis using specific antibodies was performed to examine protein expression in whole cell lysates. Representative images from more than three independent experiments are shown.

The mTOR regulates protein synthesis and cell growth by sensing cellular signals. We next examined the effects of pemetrexed and simvastatin on mTOR-dependent autophagic pathways in MSTO-211H and A549 cells by immunoblot analysis. As shown in Figure 3A–3C, pemetrexed and simvastatin cotreatment increased AMPK phosphorylation and a concomitant decrease in AKT and mTOR phosphorylation, which induced autophagy and the expression of autophagy-related proteins in a dose- and time-dependent fashion. These data indicated that pemetrexed and simvastatin cotreatment induces AMPK- and AKT-mediated mTOR-dependent autophagy in malignant mesothelioma and NSCLC cells.

### Autophagic activators attenuate apoptosis induced by pemetrexed and simvastatin cotreatment

**Figure 4:**
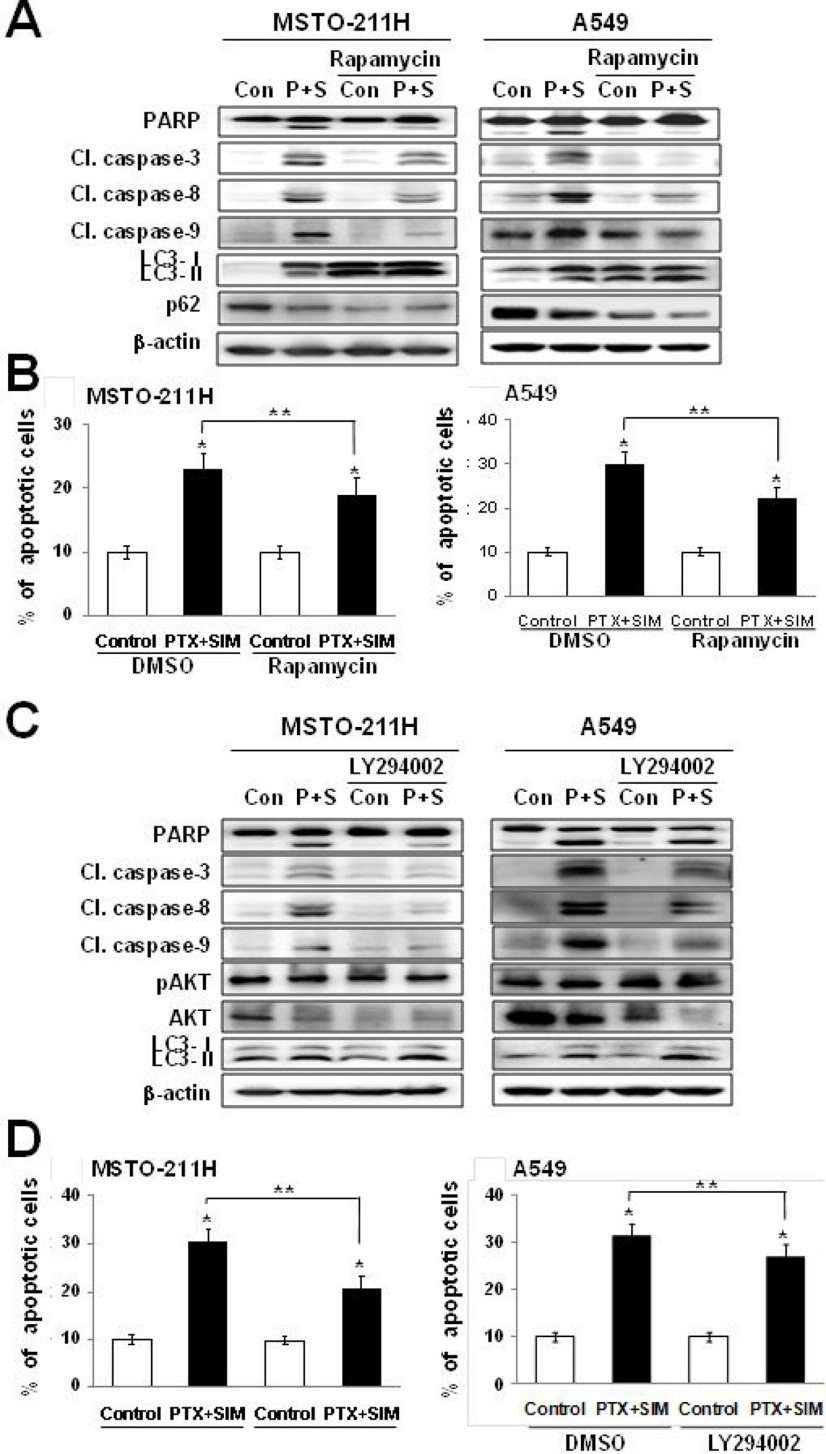
Activation of autophagy decreases pemetrexed and simvastatin-induced apoptosis **A.** Cells were treated with 1 μM pemetrexed and 5 μM simvastatin in the absence or presence of 20 nM rapamycin. The cell lysates were subjected to 12% SDS-PAGE to measure the expression of the indicated proteins. **C.** Cells were treated with 1 μM pemetrexed and 5 μM simvastatin in the absence or presence of 10 μM LY294002. The cell lysates were subjected to 12% SDS-PAGE to measure the expression of the indicated proteins. **B.** and **D.** Apoptosis was evaluated as described in Figure 1C. The data represent the mean ± SD of three independent experiments. *p < 0.01 compared with the control, ^##^*p* < 0.05 compared with the pemetrexed and simvastatin group.

Chemicals that inhibit mTOR activity via AMPK activation or AKT inhibition have been associated with the induction of autophagy in cancer cells [26, 27]. Therefore, we investigated whether suppression of mTOR function by rapamycin or LY294002 alters apoptosis induced by the pemetrexed and simvastatin cotreatment. Our results showed that LC3-II levels increased and p62 levels decreased in MSTO-211H and A549 cells cotreated with pemetrexed, simvastatin, and rapamycin. Both rapamycin and the pemetrexed–simvastatin combination also decreased the expression of cleaved PARP, caspase-3, -8, and -9 (Figure 4A). Following similar protocols, we next demonstrated that pemetrexed, simvastatin, and LY294002 cotreatment also increased LC3-II levels (Figure 4C). Annexin V staining showed that rapamycin or LY294002 activation of pemetrexed and simvastatin-induced autophagy results in significantly attenuated apoptosis compared to combination treatment in MSTO-211H and A549 cells (Figure 4B and 4D). Together, these findings indicate that AKT-mediated inhibition of mTOR by rapamycin or LY294002 attenuates apoptosis induced by pemetrexed and simvastatin combination in malignant mesothelioma and NSCLC cells.

### Autophagic inhibitors augment apoptosis induced by pemetrexed and simvastatin cotreatment

To investigate the relationship between pemetrexed and simvastatin-induced apoptosis and autophagy, cells were cotreated with pemetrexed and simvastatin using one of the four autophagic inhibitors [28, 29]: (i) 3-methyladenine (3-MA), a class III PI3 kinase inhibitor; (ii) ATG5 siRNA, an inhibitor of ATG5–12 formation; (iii) bafilomycin A, a specific lysomal vacuolar-type H^+^-ATPase pump inhibitor that blocks the fusion of autophagosomes and lysosomes; and (iv) E64D/pepstatin A, an inhibitor of lysosomal enzymes.

**Figure 5:**
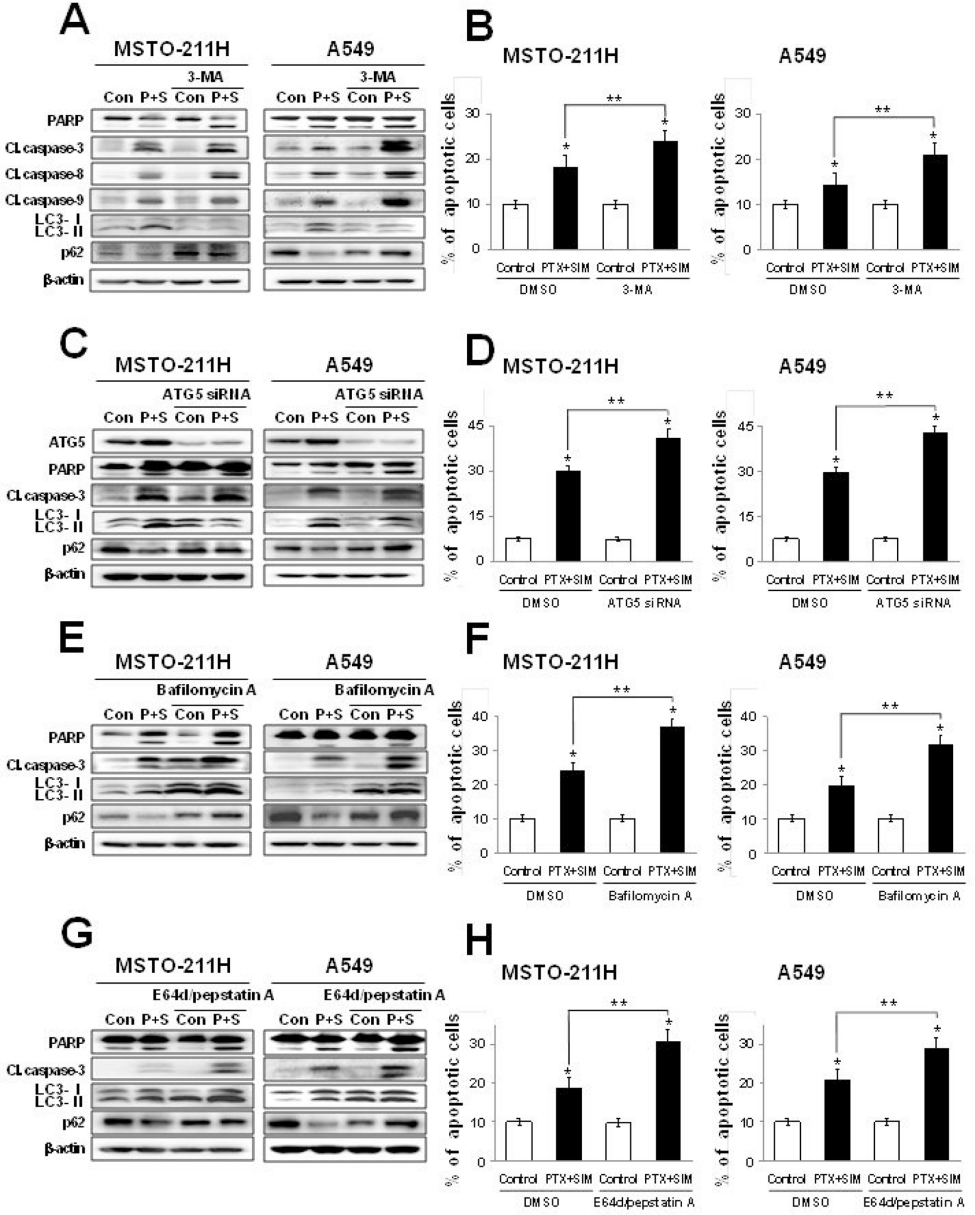
Inhibition of autophagy increases pemetrexed and simvastatin-induced apoptosis Cells were treated with 1 μM pemetrexed and 5 μM simvastatin in the absence or presence of the autophagy inhibitors 1 mM 3-MA **A.** ATG5 siRNA **C.** 50 nM bafilomycin A **E.** and 10 μM E64d/pepstatin A **G.** The cell lysates were subjected to 12% SDS-PAGE to measure the expression of indicated proteins. **B, D, F. and H.** Apoptosis was evaluated as described in Figure 2A. The data represent the mean ± SD of three independent experiments. *p < 0.01 compared with the control, ^##^*p* < 0.05 compared to the pemetrexed and simvastatin group.

Because 3-MA inhibits early autophagy events, we assessed its impact on autophagy flux when combined with pemetrexed and simvastatin. We found that this treatment decreased LC3-II levels and increased p62 levels in MSTO-211H and A549 cells. Both 3-MA and the pemetrexed–simvastatin combination also increased the expression of cleaved PARP, caspase-3, -8, and -9 (Figure 5A). Annexin V staining showed that 3-MA inhibition of pemetrexed and simvastatin-induced autophagy significantly enhances apoptosis compared to pemetrexed and simvastatin combination treatment in MSTO-211H and A549 cells (Figure 5B). To rule out non-selective effects of chemical inhibitors, we then examined how pemetrexed and simvastatin-induced autophagy is affected in ATG5 siRNA-transfected cells. ATG5 silencing accelerated apoptosis and decreased autophagy flux, as was indicated by both an increase in the cleaved forms of caspase 3 and PARP and a decrease in LC3-II levels compared to cells expressing control scrambled siRNA (Figure 5C). In addition, a greater proportion of apoptotic cells were induced by ATG5 siRNA and the pemetrexed-simvastatin combination than cells cotreated with pemetrexed and simvastatin (Figure 5D).

Many autophagy inhibitors act on post-sequestration steps and are known to cause autophagosome accumulation [30]. We found that inhibiting autophagy using bafilomycin A increased pemetrexed and simvastatin-induced cleavage of PARP, caspase-3, -8, and -9. Importantly, bafilomycin A promoted the accumulation of LC3-II, confirming a functional requirement for lysosomes in pemetrexed and simvastatin-induced autophagy (Figure 5E). We next demonstrated that the protease inhibitors E64D/pepstatin A caused the accumulation of LC3-II (Figure 5G). Bafilomycin A or E64D/pepstatin A inhibition of pemetrexed and simvastatin-induced autophagy also increased the proportion of apoptotic MSTO-211H and A549 cells (Figure 5F and 5H). Collectively, our data reveal that autophagic inhibitors provide an positive regulatory signal for pemetrexed and simvastatin-induced apoptosis.

### Interaction between pemetrexed and simvastatin suppresses tumor growth in lung cancer xenografts

**Figure 6:**
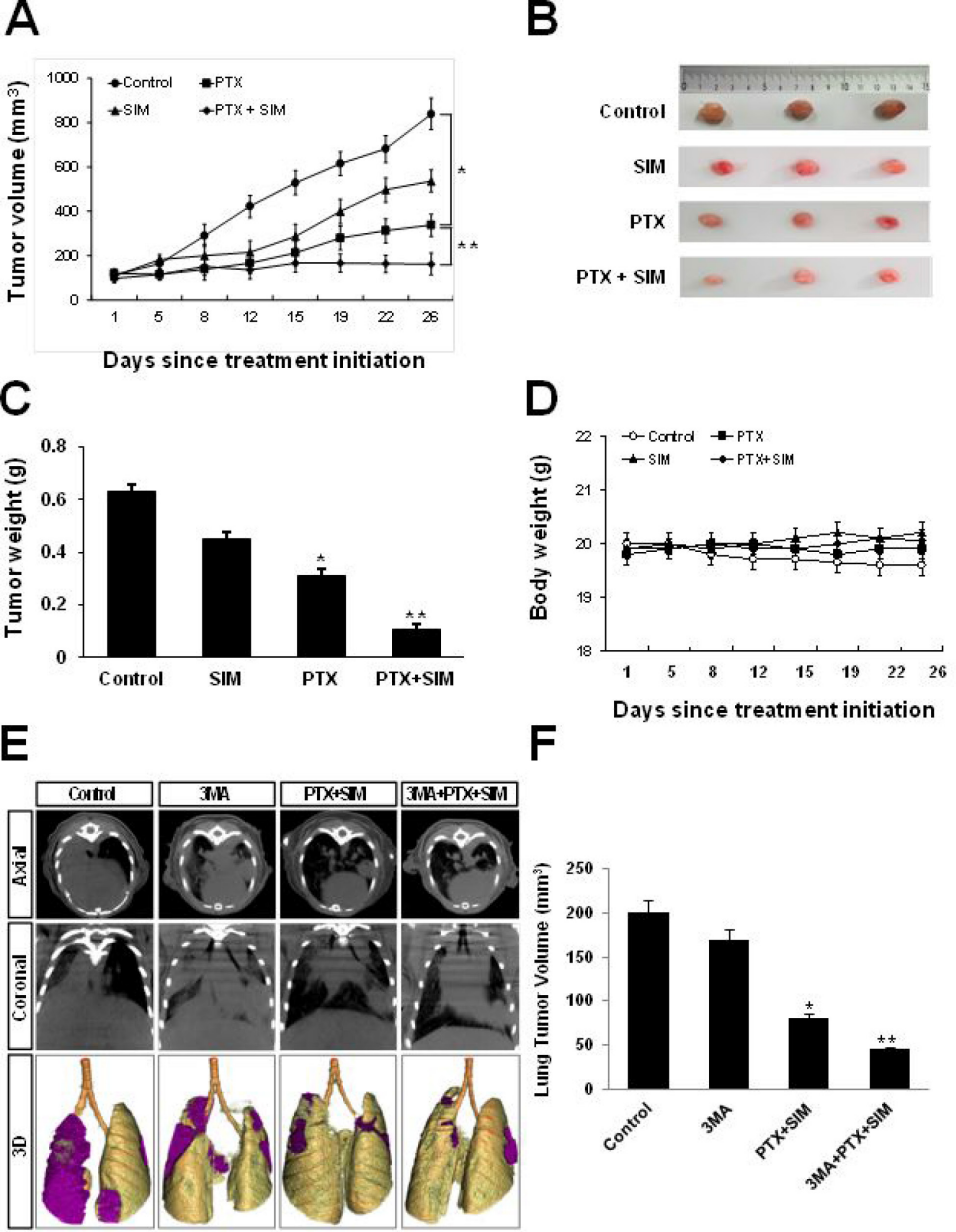
Inhibitory effects of pemetrexed and simvastatin on xenografts and growth inhibitory effects of 3-MA on orthotopics **A.** Athymic nude mice were injected (s.c.) with 2 × 10^6^ A549 cells (0.2 mL cell suspension) in both hind legs. Mice were randomly assigned to one of the following experimental groups of (*n* = 5 each) when the implanted tumors reached a volume of 90–130 mm^3^: no treatment, pemetrexed (100 mg/kg, i.p., once a week), simvastatin (20 mg/kg, by oral gavage, once every day for 5 days), and the combined pemetrexed and simvastatin treatment. Tumors were measured 3 times per week. Tumor volume was estimated using the formula: volume = *L* × *W*^2^/2. Points, mean of 5 animals; bars, SD. *p < 0.01 compared with the control, ^*^*p < 0.05 compared to the pemetrexed group. **B.** At the end of the experiment, tumor tissues were excised from mice. Representative tumor tissues from all groups are shown. **C.** The tumors were weighed. The data represent the mean ± standard deviation (SD) of 5 animals. *p < 0.05 compared to the control, ^*^*p < 0.01 compared to the control. **D.** Body weights of mice were not significantly different among all groups. **E.** Micro-CT images obtained using an orthotopic mouse model. Micro-CT images of tumors in mice in the control, pemetrexed plus simvastatin, 3-MA alone, and 3-MA and combination of pemetrexed and simvastatin groups at the indicated weeks after an intrathoracic injection of a suspension of A549 cells. Each tumor scanned using micro-CT was reconstructed to three-dimensional images (axial, coronal, and 3D). **F.** Quantitative analysis of the tumor diameter using micro-CT imaging. Data represent the mean ± standard error of mean (SEM) value of 5 animals in each group. *p < 0.05 compared to the control, **p < 0.01 compared to the control.

To demonstrate the inhibitory effects of pemetrexed and simvastatin on tumor growth in xenografts, we performed an antitumor study using athymic nude mice injected (s.c.) with A549 cells. As shown in Figure 6A, weekly pemetrexed (100 mg/kg) and daily simvastatin (20 mg/kg) cotreatment significantly reduced tumor growth to an extent lesser than that achieved with pemetrexed or simvastatin alone. Furthermore, mice co-injected with pemetrexed and simvastatin had significantly smaller tumor volumes compared to the groups receiving either pemetrexed or simvastatin alone (Figure 6B and 6C). The body weights of mice on subsequent days after the first injection of the drug were not significantly different among the 4 experimental groups (Figure 6D).

### 3-MA augments the inhibitory effects of cotreatment with pemetrexed and simvastatin in an orthotopic model of lung cancer

We monitored the inhibitory effects of 3-MA on the growth of lung cancer in an orthotopic mouse model by using micro-CT imaging, which is a noninvasive, quantitative, and real-time tool for monitoring tumor progression. 3-MA was injected intraperitoneally 1 h before coadministration of pemetrexed and simvastatin. Tumor response was quantified weekly by using serial micro-CT imaging. At 2 weeks after intrathoracic injection of tumor cell suspension, tumors in control and 3MA-treated mice grew by approximately 200 and 175 mm^2^, those in mice treated with a combination of pemetrexed and simvastatin grew by 75 mm^2^, whereas those in mice treated with 3MA plus pemetrexed and simvastatin remained 45 mm^2^, which were smaller than those of mice treated with a combination therapy (Figure 6E). These results are consistent with our *ex vivo* tumor data measured at the end of the experiment (Figure 6F).

## DISCUSSION

In the present study, we demonstrated that suppressing autophagy enhances the pemetrexed and simvastatin-induced apoptosis in MSTO-211H and A549 cells, whereas autophagy induction attenuates this process. These results clearly indicate that autophagy serves a prosurvival function against malignant mesothelioma and NSCLC cells. Furthermore, the combination of these two agents could potentially kill malignant mesothelioma and NSCLC cells more effectively and with fewer side effects than either drug alone, thereby providing a rationale for combining these drugs as a treatment for malignant mesothelioma and NSCLC.

Pemetrexed has demonstrated clinical potential, either alone or in combination with the platinum compounds vinorelbine and gemcitabine in a broad array of solid tumors [31]. Studies on pemetrexed's mechanism of action have shown that it inhibits cell proliferation and induces apoptosis and autophagy in cancer cells [32, 33]. An increasing body of evidence supports the existence of cross-talk between apoptosis and autophagy, including both positive and negative interactions [34–36]. In this regard, recent evidence suggests that autophagy may attenuate a drug-induced apoptotic response [37, 38]. To date, however, the molecular mechanisms that govern the interplay between autophagy and apoptosis are poorly understood.

To our knowledge, this is the first report highlighting the interplay between apoptosis and autophagy in malignant mesothelioma and NSCLC cells cotreated with pemetrexed and simvastatin. In an effort to determine whether autophagy serves a prosurvival or prodeath role in response to treatment with pemetrexed and simvastatin, we evaluated pharmacological and genetic approaches inhibiting autophagy.

In the present study, MSTO-211H and A549 cells cotreated with the autophagy inhibitors 3-MA, bafilomycin A, and E64d/pepstatin A displayed markedly increased expression of cleaved PARP, caspase-3, -8, and -9, suggesting that autophagy serves a protective role. This finding is further supported by our observation that pemetrexed and simvastatin-induced apoptosis was significantly increased in ATG5 silenced MSTO-211H and A549 cells. We also observed that pretreatment with 3-MA was associated with a decrease in LC3-II formation and an increase p62 levels, essentially reversing the effect of pemetrexed and simvastatin and blocking autophagy. p62 accumulation facilitates autophagic clearance [39], and evidence indicates that p62 levels are regulated by autophagy and accumulate in autophagy-deficient cells [40]. Since p62 accumulates when autophagy is inhibited, decreased levels can be observed when autophagy is induced. Therefore, p62 can be used as a marker of autophagy flux. We found that pretreatment with bafilomycin A and E64d/pepstatin A, which blocks later autophagosomal degradation, increased the formation of LC3-II in MSTO-211H and A549 cells after combination treatment of pemetrexed and simvastatin.

Autophagy-associated genes are under the control of mTOR, and the inhibition of mTOR by rapamycin or LY294002 enhances autophagy [41, 42]. AMPK activation negatively regulates mTOR activity and, thus, induces autophagy. Given the positive and negative regulatory roles of AMPK and AKT on mTOR activity, respectively, it is not surprising that both AKT/mTOR and AMPK/mTOR signaling mediate pemetrexed and simvastatin-induced autophagy. Cross talk occurring between these signaling pathways is not entirely clear and requires further investigation.

In summary, our novel findings demonstrate that pemetrexed and simvastatin-induced autophagy involves enhanced autophagosomal synthesis and may be a negatively regulated modulator of apoptosis in MSTO-211H and A549 cells. Therefore, autophagy serves a prosurvial function in our treated malignant mesothelioma and NSCLC cells. Consistent with these results, inhibition of autophagy can enhance the anti-cancer effects of pemetrexed and simvastatin and, thus, could be therapeutically targeted to improve the efficacy of these combination therapies. In conclusion, the adverse effects of pemetrexed should be ameliorated by a high dose of simvastatin administered as an adjuvant in cancer therapy.

## MATERIALS AND METHODS

### Materials

Roswell Park Memorial Institute medium 1640 (RPMI 1640), fetal bovine serum (FBS), and antibiotics (penicillin and streptomycin) were obtained from GIBCO BRL Co. (Grand Island, NY, USA). Pemetrexed was purchased from Toronto Research Chemicals, Inc. (Toronto, Ontario, Canada). Simvastatin, 3-(4,5-dimethyl-2-thiazolyl)-2,5-diphenyl-2H-tetrazolium bromide (MTT), propidium iodide (PI), and dimethyl sulfoxide (DMSO) were purchased from Sigma-Aldrich (St. Louis, MO, USA). Primary antibodies against caspase-3, caspase-8, and caspase-9, poly(ADP-ribose) polymerase (PARP), and AKT were obtained from Santa Cruz Biotechnology (Santa Cruz, CA, USA). Antibodies against AMPK, mTOR, Beclin, ATG5, LC3, p62/SQSTM1, and β-actin were purchased from Cell Signaling Technology (Beverly, MA, USA). 3-Methyl adenine (3-MA), bafilomycin A, and E64D/pepstatin A were purchased from Sigma-Aldrich. Anti-rabbit IgG-conjugated horseradish peroxidase (HRP) antibodies and enhanced chemiluminescence (ECL) kits were purchased from Amersham Pharmacia Biotech (Buckinghamshire, England, UK).

### Cell culture and viability test

MSTO-211H cells were purchased from the American Type Culture Collection (ATCC), Manassas, VA, USA, and A549 human lung cancer cells were obtained from the Korean Cell Line Bank (KCLB), Seoul, Korea. These cell lines were grown in RPMI 1640 containing 100 units/mL penicillin, 0.1 mg/mL streptomycin, and 10% FBS. All cell lines used in the study were authenticated by the ATCC and KCLB using STR-PCR analysis. The cells were incubated in a humidified atmosphere of 5% CO_2_ in air at 37°C and maintained in log phase growth. Cell viability was determined by spectrophotometrically measuring the mitochondrial conversion of MTT to formazan. After cells were treated with the specified drugs, MTT was added to the cell suspension for 4 h. After three washes with phosphate-buffered saline (PBS; pH 7.4), the insoluble formazan product was dissolved in dimethyl sulfoxide (DMSO). The optical density (OD) of each well was measured using a microplate reader (Titertek Multiskan; Flow Laboratories, North Ryde, New South Wales, Australia) at 590 nm. The OD resulting from formazan production in control cells was considered as 100% cell viability. All other measurements were expressed as a percentage of the control cell value.

### Electrical cell-substrate impedance sensing system (ECIS)

ECIS experiments were conducted using an Applied Biophysics Model ECISZθ instrument (Applied Biophysics, Troy, NY, USA). Briefly, cells (7 × 10^4^/well) were plated on collagen type I-coated 8W10E+ electrode arrays (Applied Biophysics, Troy, NY, USA), and adherence was ensured; the cells were allowed to form a monolayer for 5 h. After washing with PBS, the cells were treated with different concentrations of simvastatin in the presence of pemetrexed and resistance was measured. During the experiments, cultures were maintained at 37°C and 5% CO_2_ in air.

### Annexin V assay for the assessment of apoptosis

MSTO-211H and A549 cells undergoing early/late apoptosis were analyzed by annexin V-FITC and PI staining. Cells in the exponential growth phase (2.5 × 10^5^ cells) were seeded in 35-mm^2^ dishes. They were left untreated or incubated with specified drugs for the indicated times at 37°C. Both adherent and floating cells were collected and analyzed with the annexin V assay according to the manufacturer's instructions. Pelleted cells were briefly washed with PBS and resuspended in annexin-binding buffer. They were then incubated with annexin V-FITC and PI for 15 min at room temperature. After incubation, the stained cells were analyzed using a fluorescence-activated cell sorting (FACS) Calibur system equipped with Cell Quest software (Becton Dickinson, San Jose, CA, USA).

### Transmission electron microscopy (TEM)

Electron microscopy and morphometric analysis were performed as described previously [26]. Cells were fixed for 1 h with ice-cold 2.5% glutaraldehyde in 0.1 M cacodylate buffer, and then post-fixed in 1% osmium tetroxide before embedding in Epon. TEM was performed with a Philips CM10 at 80 kV on ultra-thin sections (100 nm on 200 mesh grids) stained with uranyl acetate and counterstained with lead citrate.

### Transient transfection

Adenoviral GFP-LC3B was kindly provided by Dr. Xiao-Ming Yin (University of Pittsburgh School of Medicine, Pittsburgh PA). After the cells were washed with OPTI-MEM medium (Invitrogen), DNA was transfected into cells using Lipofectamine^TM^ 2000 reagent according to the manufacturer's protocol (Invitrogen, Carlsbad, CA). After incubation for 4 h, the medium was exchanged with a complete medium containing 10% serum and antibiotics. The cells were incubated for 24 h and treated as indicated in the figure legends. Images were obtained using a confocal microscope (Olympus, FluoView™ FV1000).

### Acridine orange staining

Autophagy is characterized by the formation and promotion of acidic vesicular organelles (AVOs). In acridine orange-stained cells, the cytoplasm and nucleus fluoresce bright green and dim red, whereas acidic compartments fluoresce bright red or orange as described previously [27]. After drug treatment, 5 μg/ml acridine orange (Invitrogen, A1301) was added to the culture medium and cells were incubated at 37°C for 15–30 min. The cells were then trypsinized, washed twice with cold PBS, and observed under a confocal microscope. Fluorescence imaging was performed using a blue bandpass filter with 490 nm excitation and the fluorescence of the green and orange channels was recorded and merged.

### Western blotting

Cells were harvested and lysed using a radioimmunoprecipitation assay buffer (50 mM Tris-Cl [pH, 7.4], 1% NP40, 150 mM NaCl, 1 mM EDTA, 1 mM phenylmethylsulfonyl fluoride, 1 μg/mL of aprotinin and leupeptin each, and 1 mM Na_3_VO_4_). After centrifugation at 12,000× *g* for 30 min, supernatant was collected, and the protein concentration was determined by the Bradford method (Bio-Rad Protein Assay). Equal amounts of protein were separated using 12% sodium dodecyl sulfate-polyacrylamide gel electrophoresis (SDS-PAGE) under reducing conditions and were subsequently transferred to nitrocellulose membranes. The membranes were blocked with 5% skim milk in TBS-T (25 mM Tris [pH, 7.6], 138 mM NaCl, and 0.05% Tween-20) for 1 h and probed with primary antibodies (at 1:1000–1:5000). After washing, the membranes were further incubated with a HRP-conjugated secondary antibody (at 1:2000–1:10,000). Immunoreactive signals were detected using an ECL detection system.

### Gene silencing

Pooled small interfering RNA (siRNA) oligonucleotides against ATG5 were purchased from Cell Signaling Technology. Cells were seeded, and after 24 h, they were transfected with 100 nM pooled oligonucleotide mixture by using Lipofectamine2000 (Invitrogen) according to the manufacturer's protocol. Twenty-four hours after transfection, media were removed and cells were treated with the indicated drugs. Gene silencing efficacy by siRNA was assessed by western blot analysis.

### Tumor xenograft studies in nude mice

Five- to six-week-old BALB/c athymic nude mice (Charles River, Japan) were housed in cages with HEPA-filtered air (12-h light/dark cycle) and had *ad libitum* access to food and autoclaved water. A549 cells were injected subcutaneously (s.c.) into both hind legs of each mouse. Mice were randomly assigned to one of 4 experimental groups (*n* = 5 each) when the implanted tumors reached a volume of 90–130 mm^3^. Each group was monitored until tumors reached a volume of 1,300 mm^3^ or for 26 days. All procedures were performed in accordance with our institutional animal care and use policies.

### Tumor orthotopic studies in nude mice

We intraperitoneally injected 20 nude mice with thiopental sodium (0.08 mL/kg of body weight) to induce anesthesia; subsequently, the mice were placed in the right lateral decubitus position. Then, 50 μL of A549 single cell suspension (1.5 × 10^6^) prepared using the 1-mL injector was rapidly inoculated percutaneously into the upper margin of the sixth intercostal rib on the left anterior axillary line to a depth of about 5 mm, and then, the needle was promptly removed. The mice were maintained in the left lateral decubitus position after injection and were observed until complete recovery.

### Micro-CT imaging analysis

Twenty mice with lung cancer underwent micro-CT scanning weekly after cell inoculation. Whole lungs were scanned for detection of tumors at 20× magnification. Each tumor scanned using micro-CT was reconstructed to three-dimensional images (axial, coronal, and 3D). Tumor size was evaluated using an imaging software (Xelis; Infinitt, Seoul, Korea). The diameter of the tumor was defined as the maximum diameter of the tumor in a 2D plane. The tumor volume was evaluated using a volume analysis software (VGStudio MAX, Heidelberg, Germany). Small tumors were not included in data analysis because of the inability to measure the size of these tumors.

### Statistical analysis

Each experiment was performed at least 3 times, and all values were expressed as the mean ± SD of triplicate samples. The Student's *t*-test was used to determine the statistical significance. Values of *p* < 0.05 were considered statistically significant.
